# Toward a Predictive Understanding of Earth’s Microbiomes to Address 21st Century Challenges

**DOI:** 10.1128/mBio.00714-16

**Published:** 2016-05-13

**Authors:** Martin J. Blaser, Zoe G. Cardon, Mildred K. Cho, Jeffrey L. Dangl, Timothy J. Donohue, Jessica L. Green, Rob Knight, Mary E. Maxon, Trent R. Northen, Katherine S. Pollard, Eoin L. Brodie

**Affiliations:** a Departments of Microbiology and Medicine, New York University School of Medicine, New York, New York, USA; b The Ecosystems Center, Marine Biological Laboratory, Woods Hole, Massachusetts, USA; c Stanford Center for Biomedical Ethics, Stanford University, Palo Alto, California, USA; d Department of Biology and Howard Hughes Medical Institute, University of North Carolina, Chapel Hill, North Carolina, USA; e Department of Bacteriology, Great Lakes Bioenergy Research Center and Wisconsin Energy Institute, University of Wisconsin—Madison, Madison, Wisconsin, USA; f Biology and the Built Environment Center, University of Oregon, Eugene, Oregon, USA; g Department of Biology, Institute of Ecology and Evolution, University of Oregon, Eugene, Oregon, USA; h Departments of Pediatrics and Computer Science & Engineering, and Center for Microbiome Innovation, University of California San Diego, La Jolla, California, USA; i Biosciences, Lawrence Berkeley National Laboratory, Berkeley, California, USA; j Division of Biostatistics, Gladstone Institutes and Institute for Human Genetics, Institute for Computational Health Science, University of California, San Francisco, California, USA; k Earth and Environmental Sciences, Lawrence Berkeley National Lab, Berkeley, California, USA; l Department of Environmental Science, Policy and Management, University of California, Berkeley, California, USA

## Abstract

Microorganisms have shaped our planet and its inhabitants for over 3.5 billion years. Humankind has had a profound influence on the biosphere, manifested as global climate and land use changes, and extensive urbanization in response to a growing population. The challenges we face to supply food, energy, and clean water while maintaining and improving the health of our population and ecosystems are significant. Given the extensive influence of microorganisms across our biosphere, we propose that a coordinated, cross-disciplinary effort is required to understand, predict, and harness microbiome function. From the parallelization of gene function testing to precision manipulation of genes, communities, and model ecosystems and development of novel analytical and simulation approaches, we outline strategies to move microbiome research into an era of causality. These efforts will improve prediction of ecosystem response and enable the development of new, responsible, microbiome-based solutions to significant challenges of our time.

## EDITORIAL

Now well into the 21st century, the Human Genome Project fades from our rearview mirror but its lasting impact extends far into our future (1). Massively parallel DNA sequencing platforms plus significant technological advances derived from this previous international, public, and private initiative continue to drive economic development and numerous paradigm shifts across domains of the biological, physical, and social sciences. Foremost among these paradigm shifts has been the realization that our species, *Homo sapiens*, is at least as microbial as human in terms of cell numbers (2) and much more so in terms of genetic potential (3). The subsequent initiative to sequence our bodies’ “second genome,” represented by the NIH-funded Human Microbiome Project and its European equivalent, Meta-HIT, has catalyzed numerous discoveries and sparked interest in identifying the contributions of our microbiota to our health, development, behavior, and emotions (summarized in Table 1 of reference 4). As a result of this initiative and our anthropocentric tendencies, the term “microbiome” is now becoming a familiar concept to the general public and serves as a nucleation point for academic and industrial efforts aiming to uncover hidden microbial roles in health and disease and to discover microbiome-based interventions. If our efforts are successful, their societal and economic impacts will likely be substantial and accompanied by both philosophical debate and ethical considerations.

We often overlook the fact that the concept and impact of the “microbiome” extend far beyond the human body. In fact, microorganisms have populated, dominated, and shaped our planet and its inhabitants for over 3.5 billion years. Plants and multicellular animals (*Metazoa*) first emerged ~800 million and ~700 million years ago, respectively. Modern humans have existed for roughly only 250,000 years, and are thus merely a recently emerged twig in the tree of life. It is perhaps not surprising that single-celled microorganisms, the pioneers of life on Earth, played critical roles in the evolution and functioning of all other living organisms (5). Like a modern-day corporation, most eukaryotes have outsourced (or, more accurately, insourced) several key functions to bacteria (6). The mitochondrion that functions as a cellular power plant in eukaryotes evolved from once-free-living bacteria that were engulfed; similarly, the chloroplast that is the center of photosynthesis in plants was likely derived from one or more free-living bacteria. This intermingling of genes and functions across the tree of life continues, allowing multicellular organisms to adapt more rapidly to new environments, using the versatility of their microbial partners (7–15). The ubiquity of microorganisms and their breadth of impact on the habitability of our planet have prompted musings of what life would be like without them (16). However, unlike “germfree” animals or plants in the confines of the laboratory, the health of the planet’s environment and that of its inhabitants are absolutely dependent on their microbial partners.

## MICROBES DROVE THE FORMATION OF OUR BIOSPHERE

**FIG 1:**
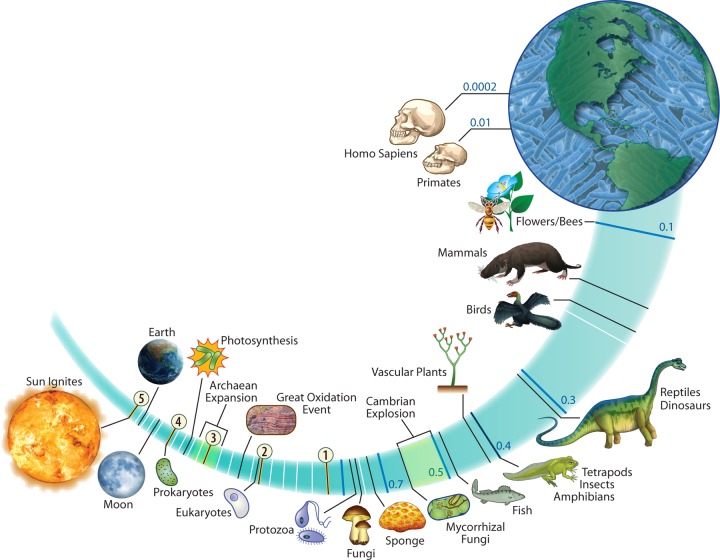
Microbial landmarks in the evolution of our biosphere. Adapted from original artwork of Mariana Ruiz Villarreal (https://commons.wikimedia.org/wiki/File:Timeline_evolution_of_life.svg).

So how did we get here? Stepping back approximately 2.5 to 2.3 billion years, we observe the Great Oxidation Event, a cataclysmic shift in the oxidation-reduction status of our planet that can be seen and traced in the geologic record, including global iron deposits (17). What was initially a nonoxidizing atmosphere, dominated by methane, hydrogen sulfide, and carbon dioxide, flipped (in geologic time) to an environment with abundant molecular oxygen. This flip was mediated by the rise of microorganisms capable of oxygenic photosynthesis, ancestors of today’s *Cyanobacteria* (18), eliminating countless oxygen-sensitive microorganisms and resulting in one of Earth’s most significant mass extinctions. However, the energy available from oxygen-coupled redox reactions (aerobic respiration) was significantly greater than the previous anaerobic lifestyles and allowed rapid diversification of new functions in a period termed the “archaean genetic expansion” (19). This period of energetic adaptation led to species diversification that was the precursor to the evolution of multicellular organisms and ultimately, plants, animals, and the remainder of the tree of life. As such, the planet’s collective microbial ancestors facilitated the formation of the biosphere as we know it (Fig. 1).

While microorganisms were initially viewed as a curiosity to be seen under the rudimentary microscopes of Anton van Leeuwenhoek, they are now appreciated as the “biogeochemical engines” that continue to support all life on Earth (20). Microorganisms are major drivers of the Earth’s carbon cycle. In the ocean, phytoplankton (single-celled photosynthetic bacteria and algae) drive the “biological carbon pump” and are responsible for approximately half of the global carbon fixed from the atmosphere each year, with the remainder sequestered by the Earth’s terrestrial vegetation (21). Microorganisms also perform key functions in the stabilization and recycling of this fixed carbon across our oceans and landforms. In our soils, microbes transform plant polymers and deposit their products on soil minerals, forming the basis for much of the Earth’s terrestrial carbon stocks (22). However, in the face of disturbances like the tillage of agricultural soils (23) or thawing of permafrost (24, 25), microbial activity can result in the release into the atmosphere of large amounts of carbon that has been stored for thousands of years, with a potentially positive feedback to global temperatures (26).

Nitrogen fixation is another remarkable chemical feat achieved by microorganisms. Microorganisms catalyze this energetically costly reaction at ambient temperatures and pressures, frequently forming close couplings (including symbioses) with higher organisms such as plants (27) and insects (28). Our planet’s ecosystems and inhabitants subsisted primarily on this microbially fixed nitrogen for 4 billion years until the beginning of the 20th century with the production of nitrogen fertilizer by the Haber-Bosch process (29). As transformative as this engineering process of nitrogen fixation was for the production of food on our planet, it utilizes approximately 1% of global fossil energy (30) for production of the heat and pressure needed to accomplish this feat without microorganisms.

Microorganisms provide a wide range of ecosystem functions beyond carbon and nitrogen cycling. Collectively, they purify the water in our rivers, streams, lakes, reservoirs, and aquifers, naturally controlling the flux of nutrients like nitrogen and phosphorus that can regulate the development of stable ecosystems and the establishment of complex food webs. However, detrimental events can occur when the balance of microorganisms in nature is altered because of either natural or human interventions. Microbes are sources of other greenhouse gases (including methane and nitrous oxide) that are more potent or long-lived than CO_2_. In agricultural systems, fertilizer and manure applications stimulate the microbial release of 4 to 6 Tg of nitrous oxide per year (31), while microbially produced methane associated with rice paddies and livestock production represents approximately 30% of global methane emissions (32). Nutrient runoff from agricultural, industrial, and municipal sources promotes the growth of harmful microorganisms in our waterways, for example, forming toxic algal blooms that threaten our water supplies, health, and ecosystems (33) and contributing to dead zones in our oceans (34). The disturbance of aquifer biogeochemistry due to the drilling of wells and irrigation contributed to the “largest mass poisoning of a population in history” (35), where microorganisms mobilized naturally occurring but previously immobile arsenic (36, 37).

## GLOBAL CHALLENGES OF HUMAN POPULATION GROWTH AND ENVIRONMENTAL CHANGE

**FIG 2:**
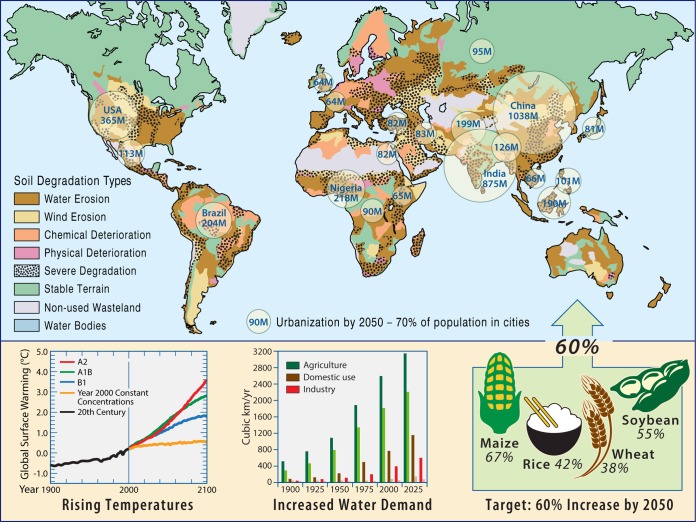
The microbiome and our changing Earth system. Population growth, urbanization, environmental degradation, and global climate change. Human-induced soil degradation based on data from reference 180; urban population by 2050 based on data from reference 181; global surface warming data based on data from reference 182; trends in global water extraction (dark color) and consumption (light color) by sector are based on data from reference 183; food yield increases required by 2050 are based on data from reference 184.

The development of the Haber-Bosch process for the production of nitrogen fertilizer that led to the advent of modern production agriculture has been described as the “detonator of the population explosion” (38). With a current population of 7.3 billion and the majority of the world’s population now residing within urban centers, we are entering an unprecedented phase in our Earth system, one that we, and our planet’s microbiomes, have never before experienced. A number of challenges arise related to sustainable production of food, energy, and chemicals to support Earth’s ever-growing human population (Fig. 2). Additionally, there is a pressing need to understand, predict, and respond to global environmental change, prevent and reverse ecosystem degradation, and manipulate the microbial origins of plant, human, and livestock diseases.

To put our current and projected future Earth system state into context, the rate of CO_2_ entering our atmosphere is unprecedented over at least 56 million years (39), demonstrating that human impacts on our planet may persist over geologic time. If this rate of emissions were to continue over the next few centuries, atmospheric CO_2_ may reach 2,000 ppmv (5 times the current concentrations), average annual temperatures would rise by 8°C, and our oceans would acidify by 0.7 pH unit (39), producing conditions not experienced on Earth since the Paleocene-Eocene Thermal Maximum ~55 million years ago. As a result, our planet’s natural biomes and those that we manage for food and fuel will likely experience conditions beyond their contemporary climate boundaries, and our current understanding of the sensitivities of their microbial components limits our ability to predict how they will respond (40).

## THE ROLE OF MICROBIOME RESEARCH TO IMPROVE HUMAN HEALTH AND RESILIENCE

The interface of the human microbiome and health is vast, and we are at the early stages of a potential scientific revolution in this area. Despite enormous progress in the provision of a stable food supply in many parts of the world, undernutrition persists for a sizable fraction of the population in many locations (41). Simultaneously, overnutrition affects a substantial, and growing, proportion of the human population, with obesity, type 2 diabetes, and other related metabolic syndromes affecting people in both developed and developing countries (42, 43). Recent studies provide evidence that particular microbiome disruptions may play important roles in malnutrition (44, 45) and obesity (46–48) and in modulating associations between diet and disease (49). Beyond the gut, the human microbiome likely affects all organs through the immune, circulatory, and nervous systems, including communicating with our brains (50) and affecting our behavior and cognitive function (51, 52).

Other emerging concepts are that a portion of the human microbiome is heritable (53) and that we have coevolved with microbes with specific properties (54). A natural extension of these concepts is that microorganisms play essential roles across the human life span, including development, maturation, reproduction, and senescence. For example, a growing body of evidence implicates microbiome perturbation during a critical window of early-life development of our immune system in the rapid increases of allergic and autoimmune conditions, including asthma, atopic dermatitis, food allergies, and inflammatory bowel disease, among others (55–58). We now appreciate that antibiotics and our increasingly industrialized lifestyles likely contribute to loss of microbes that are essential for healthy immune system development and with which our species coevolved (59, 60).

## URBANIZATION AND THE INTERSECTION OF THE HUMAN AND ENVIRONMENTAL MICROBIOMES

Urbanization is a global phenomenon occurring at unprecedented pace and scale. In 1900, only 10% of the global populations were urban dwellers. Now, for the first time in history, more than half the world’s population lives in cities. It has been projected that 70% of humanity will live in cities by 2050 (61) (Fig. 2). Cities and the buildings within them represent an unprecedented facet of human or even planetary evolutionary history. One consequence of increased urbanization is that most of the world’s people will be in regular contact with new combinations of microorganisms that thrive in urban built environments rather than the combinations of microorganisms characteristic of natural environments (62). This has prompted a new line of research to investigate the microbiology of built environments (reviewed elsewhere [63–65]). Among the emerging themes from this nascent field are that indoor microbiomes derive largely from our own bodies (66, 67) and patterns of occupancy (68, 69). In addition, several studies have demonstrated that characteristics such as surface materials and ventilation strategies influence the diversity and abundance of indoor microbial communities (e.g., 70–74). Although we currently lack sufficient mechanistic understanding to understand the importance of indoor environmental quality in terms of microbial diversity, composition, and function to our health and development, recent evidence suggests benefits of exposure to a more diverse microbiota (75–77). A critical next step is to understand the public health implications of exposure to distinct collections of microbiomes characteristic of the built environment.

## THE SOCIETAL BENEFITS OF HARNESSED MICROBIOME FUNCTIONS

Feeding our growing population is a grand challenge facing society. The last 100 years have seen great advances in increasing the amount of land that can support agricultural activity and the yield of food-grade crops per acre. The emergence of industrialized agriculture in early 20th century, improving the quantity and nutritional value of food, depended, in part, on understanding the role of nitrogen-fixing symbiotic microbes in yield and the role of the plant immune system in breeding for disease resistance. In contrast, incidents such as the “Dust Bowl,” the catastrophic wind erosion of degraded soils in the United States and Canada during the 1930s (78), and the continual emergence or spread of plant diseases (79, 80) illustrate the delicate balance between a need to intensify food production to meet population demand and the unwanted and potentially dangerous long-term consequences of altering the ecology of natural systems.

### Microbes protect our crops.

Soil microorganisms, either as individuals or as communities, both help plants acquire nutrients and help protect crops from insect pests (81) and microbial pathogens (82). Through a better understanding of these processes, we may soon be able to harness microbes to protect crops from the many microbes that cause diseases that ravage them, leading to famine (83, 84), societal upheaval, and conflict. The projected increases in population size and the desire to provide high-nutritional-quality crops to a larger fraction of the population, combined with limitations in arable land and the need to maintain or enhance ecosystem services while simultaneously increase crop yields, reinforce a need to understand the impact of plant-soil-microbe interactions on agricultural productivity. This understanding must be developed for different geographic and cropping systems to enable accurate prediction of how modern agricultural management practices impact the ecology and function of microorganisms. Determining how the interactions of microbes, plants, and soil conditions confer resistance to abiotic and biotic stress or impact nutrient availability under current or future local climate conditions is likely key to producing sufficient food for a growing population, providing the underpinnings of microbial enhancement of plant performance.

### Microbes are Earth’s “master chemists.”

The need to provide a sustainable and renewable supply of energy and chemicals is another grand challenge facing society. Microbes produce enzymes that catalyze all major biochemical transformations of inorganic and organic matter on the planet. They are also the reservoir of literally billions to trillions of genes that can ultimately be tapped for the construction of pathways to produce compounds with environmental, industrial, and pharmaceutical value. Today’s global economy is heavily influenced by humankind’s use of microbial activities, from our ancient practice of coopting yeast for brewing and baking (85), the discovery and production of antibiotics (86), microbial production of life-saving hormones such as insulin (87), and the use of nitrogen-fixing microbial inoculants to reduce fertilizer needs for food and bioenergy crops (88, 89) to the presence of enzymes in our low-temperature detergents and the recent design of microbes to synthesize fuels (90) and valuable chemicals from renewable substrates (91).

The desire to increase economic activity and affluence in emerging and developing countries is projected to create a large future demand for chemicals and fuels (92). The burgeoning demand for oil and other natural resources makes sustainable biocommodity production an attractive alternative way to meet the needs of these and other populations (93). Advances in biology, engineering, and genomics hold the promise that single species, consortia, or synthetic populations of microbes could produce alternatives to fuels or chemicals that have been derived from oil or other fossil fuels over the last 100 years (94). The microbe-based manufacturing of biocommodities could also provide numerous environmental benefits, especially if it depends on bio-based catalysts (enzymes) or sustainable and local production processes.

Several successful industrial processes use mixed microbial cultures to make food and vitamins (95, 96). A recent report of improved hydrogen, methane, or chemical production by mixed consortia (97) illustrates how knowledge of microbial activities has the potential for increasing product yield and generating fewer toxic by-products and less waste than traditional chemical processes. In addition, the use of lignocellulose or other renewable feedstocks for microbial production of fuels and chemicals can achieve reductions in net greenhouse gas emissions compared to producing the same compounds from oil or other fossil fuels (98). Other potential benefits of using microbial processes include the generally lower energy needs (temperature and pressure) for biomanufacturing and the potential for microbes to improve the efficiency of extraction or subsequent utilization of fossil fuels. Given the finite area available and our population growth-related challenges, the understanding of soil or aquatic microbial communities also has the potential for remediation and reclamation of currently contaminated environments for future use.

### Understanding and harnessing “microbial dark matter.”

Historically, our progress in harnessing specific microorganisms for societal benefit has been constrained in part by our ability to cultivate only a minor fraction of the microbial diversity we now recognize. The tools of (meta)genomics that were advanced by the human genome sequencing efforts have made possible the large-scale DNA sequencing of mixed microbial communities and have revealed that we are surrounded by “microbial dark matter.” Much like the physical sciences community has coordinated to define and understand the universe’s dark matter (99), microbiologists have embarked on a similar voyage using DNA sequencing to discover the hidden diversity and genetic potential of Earth’s microbiomes (5, 64, 100–106). As a result, we are rapidly and continually growing new branches on the tree of life (107–111), and if we are to eventually harness this new knowledge for the benefit of humankind and our planet, we must strive to define the functions contained within this vast genetic potential (112) and determine its interaction with, and regulation by, the microbiome’s local environment.

## CROSS-CUTTING CHALLENGES TO MICROBIOME-BASED INNOVATION: TECHNOLOGICAL ROADBLOCKS

**FIG 3:**
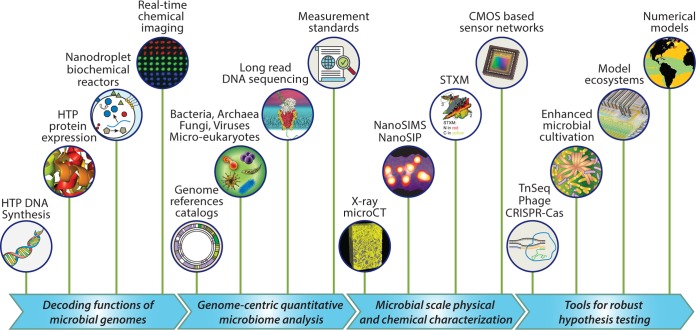
Cross-disciplinary innovations needed to advance functional understanding of Earth’s microbiomes. HTP, high throughput; STXM, scanning transmission X-ray microscopy; CMOS, complementary metal-oxide semiconductor; microCT, microcomputed tomography; TnSeq, transposon sequencing; NanoSIMS, nanoscale secondary ion mass spectrometry; NanoSIP, nanometer-scale stable isotope probing. Credits: The STXM image was adapted with permission from Remusat et al. (133), and the global ocean model depiction was adapted and reproduced with permission from Follows et al. (161).

Despite the potential to understand, predict, and harness the Earth’s critical microbiomes, several key barriers remain (Fig. 3). Just as the Human Genome Project reached across the traditional biological, physical, engineering, and social science domains to develop or respond to new technologies, next-generation advances in microbiome research must also reach beyond traditional microbiology (113).

Although there have been significant advances in our ability to obtain microbial genomic information, fundamental challenges exists regarding the scalability and portability of microbial readout technology. Even with improvements, our ability to decode the functional relevance of microbiota at appropriate scales is severely limited. Similarly, our inability to establish causality in complex microbial networks limits our ability to make informed manipulations that lead to predictable outcomes in natural systems. Without systems to predict or preempt outcomes of microbiome disturbance or manipulation, we will have limited capabilities to understand the societal impacts of this new knowledge. Success in understanding, predicting, and potentially manipulating microbiomes for societal benefit will require a broadly interdisciplinary approach; unintended consequences must be thoroughly considered.

## DECODING FUNCTIONS OF MICROBIAL GENOMES

The rate of DNA sequencing now outpaces our ability to determine gene functions by many orders of magnitude. In effect, we are transcribing countless libraries of books but have only a rudimentary understanding of the languages in which they are written. In most cases, what we hope to know is the products of these genes and their functions and how their production is regulated in nature. Across microbial genomes, there are whole families of genes possessing conserved “domains of unknown functions” that likely provide critical (but unknown) capabilities essential for microbial survival (114, 115). To identify the biological roles of these genes requires new computational approaches that decode patterns of gene covariation across environments, conditions, and genomes to predict function. We must also develop technologies for high-throughput functional determination. For example, massively parallel systems are needed so to that candidate genes can be optimized for expression, purified, or assayed *in vivo* or *in vitro*. Integrating these advances with nanoscale liquid handling (116), droplet compartmentalization of reactions (117), and high-throughput chemical imaging (118) can increase the rate of biochemical characterization of microbial genes by several orders of magnitude. Such advances will be critical to mine the genetic potential of microbes and enable a new understanding of the beneficial and detrimental aspects of microbiome function.

While obtaining genomic information has been simplified in approach, scaled in throughput, and reduced in cost, DNA sequence is a measurement of potential and not of function or activity. Other biological (macro)molecules (RNA, proteins, metabolites) provide more appropriate windows into microbial activity *in situ*, and improvements in the accuracy, integration, spatial and temporal resolution, and cost of analyzing these components will be a new frontier in microbiome research. Improved temporal resolution of microbiome gene expression (metatranscriptomics) or protein translation (metaproteomics) continues to illuminate the functional roles of individual species within complex microbial communities (119, 120), while metabolomic approaches are beginning to yield insights into the complexity of microbiome chemistry (121, 122).

Metabolomic technologies can provide critical insights into the activities of specific genes, microbes, and microbiomes, for example, when integrated with mutant libraries (123). Various forms of chromatography coupled with mass spectrometry are used for this purpose, but all are hampered by our limited ability to translate mass spectra into reliable identification of specific molecules (124). Just as the functions of many genes in a genome are unknown, most ions from mass spectrometry of microbial cultures or communities are also unknown. Efforts to develop microbiome-relevant mass spectrometry libraries would help significantly (125), supported by developments in approaches to the structural elucidation of novel metabolites. If these technical advances are accompanied by community-adopted databases and computational platforms, the broader scientific community would leverage the many parallel efforts in this area (126, 127). Because of sensitivity limitations, cost constraints, and the destructive nature of many analytical procedures, trade-offs currently exist between spatial and temporal analyses of microbiome function. The sensitivity of many existing analytical methods can limit their application to relatively large sample volumes, and for this reason, many ’omic approaches rarely sample microorganisms in the environment on the most relevant spatial or time scales.

Microorganisms exist and interact across micron-scale physical and chemical gradients, but common approaches to microbiome sampling do not capture important biological, physical, and chemical heterogeneity that is key to understanding interspecies interactions and the true environment that microbes are responding to. For example, when soil cores are homogenized to study microbial composition or activity and its relationship with soil physicochemical properties, at a human scale, this is equivalent to sampling an area of around 1,000 km^2^ (128). If microbial ecologists were to study the biological, physical, and chemical properties of the soil microbial ecosystem at the same relative scale at which plant ecologists survey these ecosystems, they would need to survey areas of 100 µm^2^, the size of soil microaggregates (128). One question is whether we need information at this scale. It appears that we might; microbe-mineral interactions at this scale are critical determinants of the storage of carbon and the retention of nutrients in our soils, and spectroscopic measurements at this scale have led to a paradigm shift in the theories of soil organic matter transformation (22). The technological barriers to studying microbiomes at the appropriate scale are immense but not insurmountable.

Discoveries at the macroscale will always be important and could be evaluated at the nano- or microscale by using targeted and potentially nondestructive approaches that are more amenable to higher spatial and temporal resolution and higher throughput. For example, infrared (IR) imaging involves the label-free detection of functional groups associated with macromolecules through acquisition of spectra that originate from vibrational frequencies characteristic of specific chemical bonds as they respond to IR light of various wavelengths. Fourier transform IR (FTIR) spectromicroscopy, a nondestructive means to monitor chemical signatures associated with microbial growth and metabolism, when combined with high-energy light sources (e.g., as generated by synchrotrons), can be deployed at or below the single-cell scale. Further developments applying nanotechnology to IR imaging many allow finer spatial resolution even without the need for synchrotron light sources (129). Although the chemical resolution of approaches like FTIR spectromicroscopy is comparatively low, as a nondestructive method, they may be coupled with destructive methods with greater chemical resolution, for example, mass spectrometry imaging based on laser or ion beam ablation (130, 131).

## PHYSICAL AND CHEMICAL CHARACTERIZATION OF MICROBIAL HABITATS

A detailed understanding of microbial interactions with their host or environment requires knowledge of the physical and chemical conditions that microbes experience directly. For example, with the exception of some aquatic environments, nearly all microbial ecosystems are associated with porous media that impact cell movement in addition to water flow and chemical diffusion (e.g., soil particles, mucous membranes, root mucilage, oral biofilms) and understanding the physical constraints on nutrient transport and communication requires physical characterization at the nanometer-micrometer scale. Approaches such as X-ray computed tomography allow detailed resolution of the physical structure of an environment at the scale of the microorganism (nanometer-micrometer) and above by the use of intact samples but currently provide limited chemical information (132). Although detailed chemical information can be obtained by methods that require thin sectioning like X-ray fluorescence, near-edge X-ray absorption fine-structure spectroscopy (133), or nanoscale secondary ion mass spectrometry (134), their destructive nature prohibits our ability to monitor the dynamics inherent to microbial systems.

At the micrometer-centimeter scale, electrochemical and optical probes have been productively used to profile gradients in pH, redox, and oxygen; however, these probes and their associated equipment are intrusive to the ecosystem and often expensive, limiting their application to only a few point measurements or limited time series, and their fragile nature makes them best suited for laboratory use. New applications of low-cost, low-power, silicon-based sensor arrays (e.g., charge-coupled device or complementary metal-oxide semiconductor) have the potential to deliver field- or lab-deployable sensor networks to monitor both the variability of environments’ physical (e.g., temperature or moisture) and chemical properties and the activity of microorganisms (e.g., nutrient transformation or respiration). Autonomous sensor networks of this form could expand the monitored scale from centimeters to kilometers, allowing microbial information to be utilized at scales relevant to gaining knowledge to understand, predict, and possibly mitigate some impacts of disturbance, such as climate change. These networks would clearly have broad applicability in water and environmental quality monitoring, agriculture, and many areas of industry.

## TECHNOLOGIES FOR ROBUST, PORTABLE, GENOME-CENTRIC ANALYSES OF MICROBIOMES

The types of global monitoring and data integration required to develop a predictive understanding of Earth’s microbiomes also require significant advances in DNA sequencing. Further reductions in cost and turnaround time, as well as improved data integration across DNA sequencing platforms and unit mobility, could allow real-time “field” studies so researchers reliably distinguish members of different microbiomes and can readily observe their dynamics. Continued transformative improvements in DNA sequencing technologies could provide systems to facilitate more robust genome-centric analyses in a manner that would allow rapid data turnaround in field-deployable units. Such approaches, if integrated with appropriate user interfaces and standardized computing platforms, could make DNA sequencing-based analysis of microbiomes as routine as a blood test or a water nitrate measurement. However, simply acquiring more sequence data does not represent a panacea. New technologies that increase sequence throughput and mobility must be accompanied by parallel advances in bioinformatics and statistics, first to ensure data quality and comparability but also to synthesize this information into biologically meaningful formats, driving the adoption of, and accessibility to, quantitative genome-centric microbiome information.

## BUILDING THE FRAMEWORK FOR MASSIVELY PARALLEL GENOME-CENTRIC QUANTITATIVE MICROBIOME ANALYSIS

*De novo* assembly of microbiome sequence data represents a massive computational burden that currently requires supercomputing facilities, and the population variation within genomes that is common to microbiomes can inhibit complete assembly. Advanced technologies that deliver long sequence reads will undoubtedly help with both of these issues; however, as we expand our investigation of microbiomes, there is a need for high-quality reference catalogs of microbial genomes. The need is not simply for more sequence information, but rather for a supporting and extensive catalog of reference genomes for which functional roles have been elucidated. Currently, the microbial gene or genome catalogs represent a minute fraction of known microbial diversity, with the entries heavily biased toward a few species and environments. Just like the targeted broadening of diversity within our human genome catalogs, initial efforts to expand microbial genome and gene catalogs have begun (135, 136). Catalogs to date have focused mostly on bacteria and archaea because of their lower genome complexity; however, critical components of most microbiomes (viruses, fungi, and other microeukaryotes) have not received the same attention. While many important microbial targets for sequencing may not be immediately culturable, approaches based on single-cell sorting and subsequent sequencing (108, 137) will be important components in building out these global microbiome references. A coordinated effort to produce and share such reference catalogs would substantially enhance the predictive value of metagenomic sequence information, while also potentially reducing the computational burden that is required for *de novo* analyses. Each of these advances drives toward a future where the computation and prediction of the functional importance of microbiome composition will be directly determined on handheld devices, enabling rapid and accurate source tracking and monitoring of microorganisms from our hospitals to our farms and oceans.

Despite transformations in our ability to decode microbial nucleic acids, analysis of the composition of a microbiome remains fraught with biases—that are largely ignored. The extreme bias introduced through cultivation of microorganisms was noted and drove the adoption of cultivation-independent approaches; however, DNA extraction alone can introduce greater variance in detected microbial abundance than the variables whose impact we wish to understand (138, 139); such observations must lead to standardization of protocols (100). However, given the variation within and between systems under study (e.g., soils, intestines, water, air, insects, plants, and even computer keyboards and cell phones), a universal nucleic acid extraction method, while researchworthy, may be unrealistic. Following nucleic acid extraction, all subsequent steps (e.g., purification, amplification, library preparation, sequencing, data analysis) introduce more unquantified uncertainty and bias that prohibit truly quantitative analyses. In the likely absence of a universally appropriate and accepted protocol, bias must be quantified, for example, by using universal standards added to samples at appropriate stages of processing. These standards would be validated by dedicated organizations such as the National Institute of Standards and Technology and supplied as components of commercial extraction kits or individually. Such a set of standards would allow the community to emulate others such as the Microarray Quality Control Consortium (140) in standardizing data generated across protocols and platforms while encouraging market diversity. It will be critical to publicly share experimental metadata and quality information alongside primary data in formats that can be easily queried and included in statistical models.

## DEVELOPING AND INTEGRATING TOOLS FOR ROBUST HYPOTHESIS TESTING

The complexity of most naturally occurring microbiomes and our inability to cultivate the majority of microorganisms naturally led to the widespread use of cultivation-independent methods to determine composition and predict function. To date, the majority of microbiome studies infer causation from correlation, particularly when more diverse microbiomes are the subject of investigation. Microbiomes, as well as being complicated, are also typically complex, with many organisms combining in a nonlinear manner to form an integrated network with emergent properties. As a result, we often lack the ability to test (i) predictions of keystone microbial species, (ii) assumptions of high functional redundancy, and (iii) the belief that microbial communities are highly resilient to disturbance. In model organisms, we can study complex genetic and metabolic networks through precise manipulation of the components. For each node in these networks (e.g., genes), we can subtract or silence, add or enhance, either individually or in combination, and observe the response of subnetworks or the entire network to these precise manipulations. This allows accurate testing of the roles of individual components in addition to their importance in the system context. Unfortunately, our ability to perform similar analysis for complex networks of microorganisms is limited.

What if we could specifically remove or inhibit a given organism, a group of organisms, or specific functions shared across many organisms and observe the response of the system as a whole? This would transition the era of microbiome study away from correlation and toward the knowledge needed to attribute causation, improve prediction, and enable precise manipulation.

Building defined microbial communities using tens to hundreds of isolated organisms is a valuable means to test the roles of individuals in low-complexity systems. However, this may be akin to building a genome *de novo* from a subset of genes to determine how the whole system functions, yet even this approach leads to surprises (115). To systematically evaluate functional roles in complex coevolved microbiome networks, subtracting organisms, observing the system’s function, and subsequently replacing organisms (or mutant variants) in a manner analogous to gene deletion and complementation would precisely define the roles of individuals and their key functions. To accomplish this full cycle will require tools that precisely inhibit specific microorganisms, an ability to cultivate (or selectively capture) more microorganisms, and the ability to rapidly develop comprehensive mutant libraries in addition to all of the aforementioned tools for monitoring the environment and the microbiome’s composition and function.

Several opportunities exist for the manipulation of a microbiome and its constituent parts in well-studied model or laboratory systems. Beginning at the population level, new technologies allow the high-throughput disruption of genes and the monitoring of their contributions to microbial fitness. High-density transposon mutagenesis coupled with high-throughput sequencing (transposon sequencing [141], for example, and its barcoded derivative random bar code transposon site sequencing [142]) allows high-throughput fitness profiling of populations of mutants cultivated under selecting conditions. These approaches have revealed the essential roles of genes with no previously known function (143), steadily increasing our view of gene essentiality (144). Alteration of individual genes, species, or functional groups in a complex microbial community could be achieved via sequence-specific gene editing or deletion with CRISPR/Cas9 delivered by phage or conjugative elements (145, 146) and the use of contractile nanotubes that can target bacteria with strain-specific activity (147). In addition, as our understanding of metabolic networks in microbiomes advances, manipulation of specific members or functional groups may be achieved through the addition or removal of substrates based on thermodynamic or kinetics-based model predictions.

The properties of the physical environment are also potentially critical determinants regulating individual fitness within microbiomes (11, 148). Consequently, the design and construction of synthetic systems with controlled physical properties such as permeability, porosity, and roughness based on natural systems will be extremely valuable in determining the key factors regulating microbiome assembly, development, stability, and activity (149–151).

Next-generation mathematical models are required to represent the complexity of microbiomes, scaling perhaps from the fundamentals of microbial electron transport (152) and the thermodynamics of microbial redox reactions (153), to genome-scale metabolic models of individuals and populations (154, 155), to microbial community function at the ecosystem (e.g., gut, soil, ocean) (156–160), and ultimately to the Earth system scale (161–163). To be useful, these models should embed the properties of microbial physiology and evolution into the physical, chemical, and biological heterogeneities characteristic of the intended length, time, and spatial scales of communities. Fully coupled models, such as those representing the microbial, environmental, and host aspects of the system (e.g., plant rhizosphere or animal intestinal tract) would allow dynamic feedbacks to be evaluated and would enlighten our understanding of the emergent phenomena (164).

## THE POTENTIAL FOR TRANSFORMATIONAL DISCOVERIES UNDER A UMI

**FIG 4:**
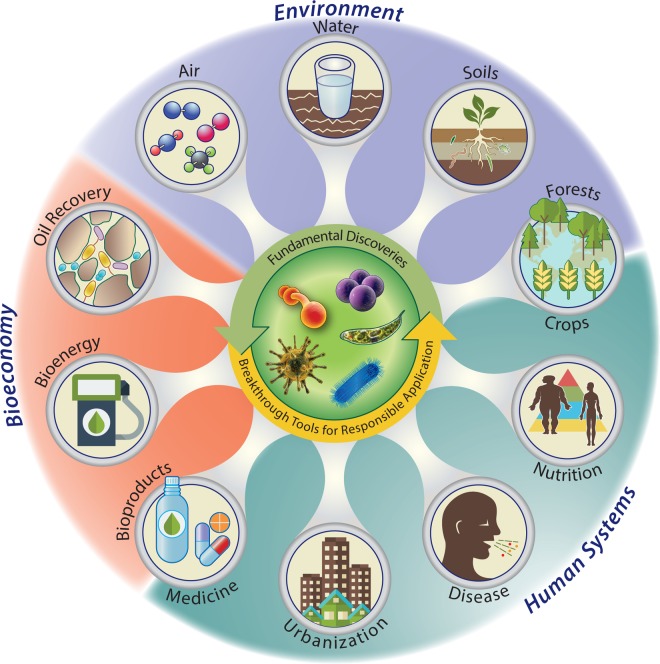
The potential impact of a unified microbiome initiative to understand and responsibly harness the activities of microbial communities.

Advancing microbiome science will require the cooperation, coordination, and collaboration of scientists and engineers from many disciplines—just like our natural ecosystems, diversity promotes productivity and stability. By extension, such efforts would likely require diversity in funding and, ideally, coordination of federal agencies, private industry partners, and philanthropic donors. For this reason, we, as a community of scientists, are one of several groups that support calls for a unified microbiome initiative (UMI) (4, 165, 166) and agree with calls for such an initiative to be built upon local leadership (103). In considering the potential value of a UMI, it is clear that improved knowledge of the activities of microbial communities can positively impact our health and that of our planet and importantly inform decision making on social and economic issues (Fig. 4)***.***

## POTENTIAL BENEFITS OF A UMI TO GROWING BIOECONOMIES

There are many examples of the profound effect of biotechnology on the medical, agricultural, and industrial economic sectors. In 2012, revenues from genetically modified organisms were ~2.5% of the United States gross domestic product, and the resulting United States National Bioeconomy Blueprint called for research and innovation to create a new bioeconomy (167). The UMI could provide knowledge to develop new microbial community applications, including designer communities for crop plants, animals, pollinator species, or rain clouds that could improve agricultural output, help mitigate the ecological and economic impact of drought, and in the case of livestock microbiomes, greatly reduce the greenhouse gas contribution of agricultural practices. The knowledge derived from UMI activity can also spur the development of sustainable methods to produce valuable bio-based fuels and commodities, improve the recovery of valuable subsurface fuels and chemicals, enable the manufacture of new bio-inspired materials, and catalyze the development of new industries that generate high-value products from renewable waste while lessening society’s reliance on fossil fuels. Achieving this goal requires an understanding of biological systems and the creation of resultant biotechnologies to benefit humankind, enhance the biosphere, and enable economic growth. UMI-enabled discoveries can have a substantial positive societal and economic impact.

## GLOBAL ETHICAL, LEGAL, AND SOCIAL ISSUES ASSOCIATED WITH A UMI AND THE POTENTIAL FOR INNOVATION

The public benefit of a UMI can be enhanced by a coordinated set of transdisciplinary, transcontinental research activities. For example, the ability to address drought resistance in crops or remediation of contaminated waters or soils needs attention by international scientific, ethical, and political experts. Recent successful examples of transcontinental research initiatives include the Human Genome Project and the International Stem Cell Initiative. Similar cooperation on the UMI could galvanize private and public funders from around the globe to collaborate on setting science priorities; provide training for scientists, legal, ethics and policy experts; harmonize international trade and intellectual property issues; and develop a suite of local, regional, and even global funding instruments to maximize societal benefits.

Given the wide-ranging potential impacts of the global microbiome, the pursuit of UMI-based discoveries and solutions must incorporate ethical and societal implications of these discoveries and their applications and consider the current biotechnology regulatory environment. However, because it has implications for human, animal, and crop health, as well as the environment more broadly, microbiome research poses novel challenges for existing regulatory frameworks (168). The gaps in current regulation mean that meeting these challenges at a pace that keeps up with microbiome science will likely require innovation in the implementation of ethical and societal implications in the scientific process.

Microbiome research and applications present unique challenges to the existing global regulatory systems (169) because traditional risk structures—the risk-benefit analyses used for traditional biotechnology products such as protein therapeutics—do not apply and because microbial communities have the potential to evolve and interact with ecological networks that cross national borders. In addition, applications of findings from microbiome research can occupy niches that are not clearly in the jurisdiction of any particular government agency. In the United States, for example, regulation by the Food and Drug Administration is product based and focuses on the safety of products for humans and animals, but probiotics and prebiotics do not fit into regulated categories. The U.S. Department of Agriculture focuses on food safety and animal and plant health but not environmental impacts of interventions that target animal or plant microbiomes. The Environmental Protection Agency regulates pollutants and toxins through the Clean Air Act and the Clean Water Act and animals, plants, and other species through the Endangered Species Act. However, these strategies do not apply to regulation or oversight of modification of naturally occurring microbial species. Recognizing that science advances may require new regulatory policies, the U.S. Government recently released a memorandum (170) to initiate a process to update the Coordinated Framework for the Regulation of Biotechnology, last revised in 1992. The aim is to coordinate and modernize the federal regulatory framework and systems that govern the vastly altered landscape of biotechnology products, including the Food and Drug Administration, the Environmental Protection Agency, and the U.S. Department of Agriculture, while attempting to reduce barriers to innovation.

Recent ethics and policy discussions regarding synthetic biology (171), genome editing (172), and approaches such as those using CRISPR-Cas systems to modify and drive the evolution of mosquito populations in the wild (173) point similarly to a need for professional self-regulation and for individual scientists to become aware of, identify, and incorporate ethical and societal considerations into actual practice (174). National-level discussions that continue to rely on the concepts of biohazard containment and risk management will likely be insufficient tools for future UMI-based research, since this work will likely provide new definitions of what is “normal,” “healthy,” or “diseased” (175).

On the other hand, a UMI provides rich opportunities to test innovations in integrating ethical and societal considerations into microbiome research, with the input of a wide range of scientific disciplines and stakeholders. One goal of such innovation could be to use research ethics consultation (176) and stakeholder engagement (177) to identify how aims and benefits of microbiome research, and thus the underlying values, can be brought into alignment with needs of relevant communities. Engaging the public in microbiome research through crowdsourcing (e.g., the American Gut Human Food Project) and citizen science (178, 179) could enhance trust in the research by transforming “the public” into stakeholders and encourage broader discussion about ethical responsibilities that would extend beyond the professional scientific community (4). It would be irresponsible to proceed with mass manipulation of microbiomes without having the structures and knowledge in place to evaluate the potential consequences.

## CONCLUDING POINTS

As was the case in other game-changing scientific initiatives (the Human Genome Project, the development of the Internet, the exploration of space), achieving the goals of the UMI requires combined expertise and technologies spanning numerous domains. The resultant discoveries and enabling technologies can provide the underpinning knowledge to develop applications within or across human and animal health, food production and safety, and the environment—all contributing to robust and sustainable bioeconomies while preserving the intrinsic value and biodiversity of our ecosystems. These applications have the potential to transform many scientific disciplines, to impact scholars in the social sciences and elsewhere, to spawn new economic opportunities, and to benefit the lives of citizens around the globe.
